# Qingke Pingchuan granules as adjuvant therapy for acute exacerbation of chronic obstructive pulmonary disease, acute exacerbation asthma, and acute bronchitis: a systematic review and meta-analysis

**DOI:** 10.3389/fmed.2026.1772299

**Published:** 2026-03-17

**Authors:** Jiaxiang Li, Jianan He, Ming Gao, Zhiyue Yu, Xiaowen Wu, Limei Geng, Huiting Hu

**Affiliations:** 1 Graduate School, Hebei University of Chinese Medicine, Shijiazhuang, Hebei, China; 2 Department of Respiratory and Critical Care Medicine, The First Affiliated Hospital of Hebei University of Traditional Chinese Medicine, Shijiazhuang, Hebei, China; 3 Department of Respiratory and Critical Care Medicine, Hebei Provincial Hospital of Traditional Chinese Medicine, Shijiazhuang, Hebei, China

**Keywords:** acute asthma exacerbation, acute bronchitis, acute exacerbation of chronic obstructive pulmonary disease, meta-analysis, Qingke Pingchuan granules

## Abstract

**Objective:**

Acute exacerbation of chronic obstructive pulmonary disease (AECOPD), Acute Exacerbation of Asthma(AEA), and acute bronchitis (AB) are known as the common acute airway inflammatory illnesses. This study seeks to systematically assess the effective rate and safety of Qingke Pingchuan Granules (QKPC) in treating these three illnesses in order to give evidence-based recommendations for therapeutic practice.

**Methods:**

Web of Science, PubMed, Cochrane Library, Elsevier ScienceDirect, CNKI, Wanfang Data, and VIP databases were all thoroughly scoured between their creation and November 2025. Leveraging the Cochrane Risk of Bias tool, two reviewers independently sifted through the reports, retrieved study data, and assessed the methodological rigor. RevMan 5.4 and Stata 17.0 software were used for statistical analyses, and effect sizes were reported as RR or MD with 95% CIs. The Cochrane Q test and the I^2^ statistic were used to evaluate heterogeneity.

**Results:**

21 RCTs with 2087 individuals were incorporated into this study. The findings demonstrated that QKPC in conjunction with conventional treatment considerably improved clinical outcomes, including overall response rate, FEV₁%, and FVC, as compared to conventional Western medicine alone. Additionally, it decreased the inflammatory factor (CRP). QKPC significantly improved the CAT score, mMRC score, 6MWT distance, and PaO₂ for AECOPD-specific outcomes (all *p* < 0.05). It decreased serum IgE and increased PEF for AEA. It reduced the time it took for AB to resolve their cough, and also reduced TNF-*α* and IL-1β levels. The incidence of adverse events (mainly gastrointestinal reactions and skin rashes) did not differ significantly between the two groups (RR = 0.73, 95% CI [0.49, 1.09]), with no significant abnormalities in liver or kidney function observed. The results’ robustness was validated by sensitivity analysis, and publication bias adjustment had no effect on the importance of the main conclusions.

**Conclusion:**

In patients with AECOPD, AEA, and AB, QKPC in conjunction with traditional Western medicine treatment can dramatically enhance clinical efficacy, lung function, and inflammatory status without raising the risk of adverse reactions. For these acute airway inflammatory illnesses, it is a safe and effective adjuvant therapy alternative that indicated to significantly improve symptoms, lung function, and quality of life in individuals with AECOPD.

**Systematic review registration:**

Unique Identifier (CRD420251229191), (https://www.crd.york.ac.uk/PROSPERO/view/CRD420251229191).

## 1 Introduction

Worldwide, acute airway disorders are common and clinically significant ailments. Among these, acute bronchitis (AB), Acute Exacerbation of Asthma (AEA), and acute exacerbations of chronic obstructive pulmonary disease (AECOPD) are common respiratory crises that are usually marked by an abrupt worsening of symptoms like wheezing, coughing, and sputum production (1, 2). Acute coughing is common with AECOPD and aggravates mucus hypersecretion and airway irritation (3); AEA is primarily driven by airway inflammation and bronchial hyperresponsiveness, manifesting clinically with cough, wheeze, and dyspnea (4); and AB is defined by abrupt, intense cough, which is often accompanied by upper respiratory tract inflammation (5, 6). Airway inflammation and hyperresponsiveness are common pathogenic mechanisms during the acute phase of many illnesses, despite differences in diagnostic criteria and pathophysiological aspects (7, 8). According to Traditional Chinese Medicine (TCM), the pathogenic mechanism of phlegm-heat obstructing the lung is shared by all of them (9, 10). The goals of treating both are to eliminate heat, relieve cough, and remove phlegm (9, 11).

With high incidence, frequent recurrence, and substantial negative impact on patients’ quality of life, these acute airway conditions collectively impose a heavy global disease burden (12–14). From an epidemiological perspective, airway diseases have become a major global public health priority. AECOPD imposes substantial economic burdens across the Asia region and around the world (15). A population-derived survey across nine countries reported a chronic obstructive pulmonary disease (COPD) prevalence of approximately 6.2%, with 46% of patients experiencing acute exacerbation annually (16). Globally, asthma, another highly prevalent chronic respiratory disease, also imposes a significant burden during AEA (17). Studies indicate that 6.5% of adults with mild asthma experience at least one AEA per year (18, 19). For AB, an acute cough constitutes the primary source of its burden (20). The natural course of an acute cough can last several weeks: approximately 93% of patients with upper respiratory tract infections (URTI) develop cough, of whom 78% experience cough for ≥1 week, and a small proportion for up to 10 weeks (21, 22).

Significant limitations still exist even though the current care of acute exacerbations of all three illnesses tries to quickly reduce inflammation, remove airway obstruction, and alleviate symptoms. Osteoporosis, immunosuppression, and hyperglycemia are among the side effects of long-term or recurrent corticosteroid use (23); bronchodilators provide only symptomatic relief of bronchospasm without addressing underlying inflammatory progression (24); and in AB, clinicians often face the problem of antibiotic overuse (25). Consequently, complementary treatment approaches are desperately needed. When it comes to the diagnosis of airway illness, traditional Chinese medicine (TCM) has proven to be very beneficial (26, 27). There is growing evidence that TCM therapies can improve patients’ quality of life, decrease adverse medication reactions, and increase therapeutic efficacy (28, 29).

**Figure 1:**
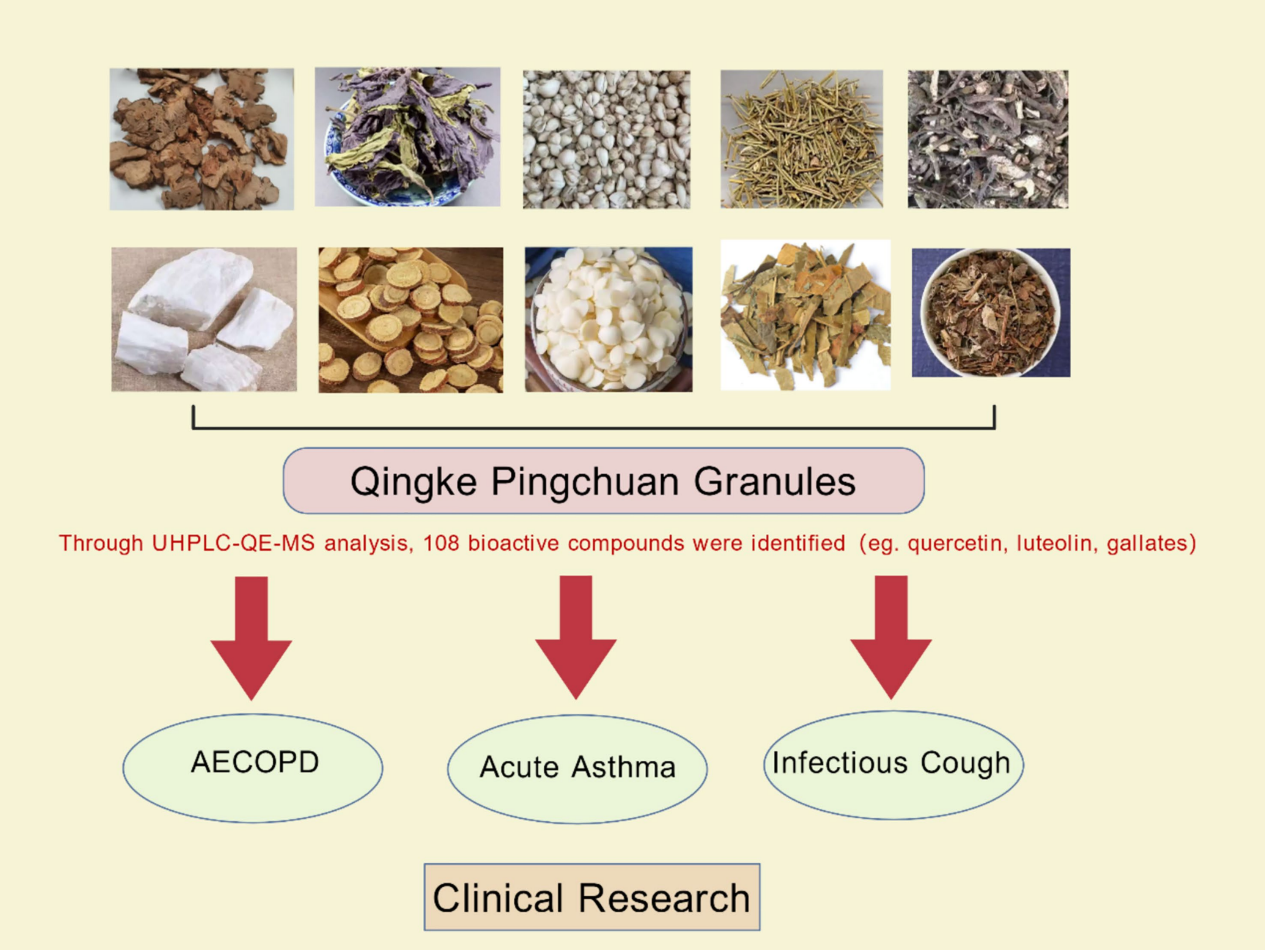
Herbal composition of Qingke Pingchuan granules.

The traditional prescription Maxing Shigan Tang (Ma Xing Shi Gan Tang) is the source of Qingke Pingchuan Granules (QKPC), a trademarked TCM formulation with drug approval number Z20040047. This remedy has long been used for acute respiratory ailments; it was detailed in Shanghan Lun -Treatise on Cold Damage Disorders (30, 31). Drug composition is shown in Figure 1. 108 bioactive compounds, including quercetin, luteolin, and gallic acid—molecules with established anti-inflammatory, antioxidant, and antifibrotic qualities—have been found in QKPC by contemporary pharmacological investigations employing UHPLC-QE-MS (30).

Clinically, QKPC has shown encouraging results in bronchodilation, anti-inflammatory action, and cough alleviation. Its safety profile and therapeutic value in the three acute airway diseases indicated above, however, have not yet been thoroughly assessed. In order to close this gap, the current study performs a meta-analysis of the clinical data that is currently available with the goal of elucidating the safety and effectiveness of QKPC in these acute situations and offering superior, evidence-derived practice guidance for clinical care.

## 2 Methods

### 2.1 Trial registration

The analysis was listed as CRD420251229191 with the International Prospective Register of Systematic Reviews (PROSPERO). Public link: https://www.crd.york.ac.uk/PROSPERO/view /CRD420251229191. To reporting our findings, we conducted our analysis in strict adherence to the methodological requirements for meta-analyses (32).

### 2.2 Search strategy

From the journals’ founding until November 2025, we systematically searched a number of databases, including PubMed, Web of Science, Cochrane Library, Elsevier ScienceDirect, CNKI, Wanfang, and VIP databases. For Chinese literature, we precisely searched term: Qingke Pingchuan + Qingke Pingchuan Granules and Chronic Obstructive Pulmonary Disease + AECOPD + Asthma + Acute Attack Stage of Asthma + Cough + Infectious Cough + Bronchitis. We used strategic search phrases in English: “Qingke Pingchuan,” “Qingke Pingchuan Granules,” “COPD,” “chronic obstructive pulmonary disease,” “acute exacerbation,” “asthma,” “bronchial asthma,” “cough,” “infectious cough,” and “acute cough.” For additional specifics, consult the detailed search methodology documented in Supplementary Appendix Table 1.

In addition, reference bibliographies of relevant studies and systematic reviews were manually screened to identify potentially eligible studies not captured by the electronic search. Screening and Full-Text Evaluation were conducted independently by two reviewers (Ming Gao and Jianan He) using a double-blind method to reduce screening bias. Throughout the process, reviewers were blinded to the authors, institutions, and trial results. Disagreements were resolved through coordination by a third reviewer (Yu Zhiyue).

### 2.3 Inclusion and exclusion criteria

Inclusion criteria:

a. Study design is RCTs;

- Participants with a clinical diagnosis of acute exacerbation of COPD (AECOPD), acute exacerbation asthma (AEA), or acute bronchitis (AB);
- Intervention: the experimental group received Qingke Pingchuan Granules (QKPC);
- At least one critical outcome was reported: Effective rate, adverse events, FEV₁% predicted, FVC, C-reactive protein (CRP), tumor necrosis factor-*α* (TNF-α), interleukin-1β (IL-1β), arterial partial pressure of oxygen (PaO₂), COPD Assessment Test (CAT), 6-min walk test (6MWT), modified Medical Research Council (mMRC), or peak expiratory flow (PEF).

Exclusion criteria:

- Non-RCT, conference paper, animal studies, or review studies;
- Participants with co-occurring non-airway conditions;
- use of proprietary Chinese herbal items or other traditional Chinese medicines (other than QKPC or its decoction form) in the experimental group;
- studies with conflicting or lacking results that could not be clarified or expanded upon by contacting the original authors;
- Duplicate publications.

### 2.4 Data extraction

Data extraction was performed independently by two reviewers (Gao Ming and He Jianan). Disagreements were resolved through coordination by a third reviewer (Yu Zhiyue). Extracted data included:

- first author and publication year;
- sample size, mean age, gender ratio, Smoking history and treatment duration;
- drug regimens for both experimental and control groups;
- Outcome measures: Primary outcomes: Effective rate: Calculated as (marked effective + effective)/total cases × 100%, referring to the Guidelines for Clinical Research of New Chinese Medicines (33): Marked effective = TCM syndrome score reduction ≥70%; Effective = 30% ≤ score reduction <70%; Ineffective = score reduction <30%, adverse events, FEV₁%, FVC, CRP; AECOPD-specific outcomes: PaO₂, CAT, 6MWT, mMRC; Acute asthma-specific outcomes: PEF, IgE; Acute bronchitis - specific outcomes: days to cough resolution, IL-1β, TNF-*α*.

We demonstrated our commitment to data integrity and thoroughness by proactively contacting the authors when the published data raised concerns or seemed lacking. All QKPC used in included trials were sourced from manufacturers certified under Good Manufacturing Practice (GMP), with production adhering strictly to the Chinese Pharmacopeia (2020 Edition) (34) and bearing the drug approval number Z20040047, ensuring pharmacological consistency across studies.

### 2.5 Data coding and management

Zotero 7.0.27 was used for literature management. Two reviewers (Gao Ming and He Jianan) independently evaluated the studies quality using the Cochrane Risk of Bias Tool. The development of randomized sequences, allocation concealment, participant and researcher blinding, outcome data completeness, reporting bias, and other potential biases were all part of our evaluation. Each study’s methodological quality was methodically divided into “low risk,” “high risk,” and “uncertain risk” levels. Meanwhile, we referred to the GRADE framework (35) for assessing the certainty of evidence and evaluated the quality of evidence for core outcomes by synthesizing factors including risk of bias, heterogeneity and publication bias.

### 2.6 Statistical analyses

For the statistical data analysis, we used Stata 17.0 and RevMan 5.4. RevMan 5.4 software was used to generate forest plots and funnel plots and calculate pooled effect sizes, while Stata 17.0 software was employed for sensitivity analyses and Egger’s test for publication bias. Odds ratios or risk ratios were employed to represent effect estimates; mean differences or standardized mean differences were used for continuous outcomes. For heterogeneity assessment, we employed the Q test and I^2^ statistic. Our model selection was conducted in accordance with the guidelines proposed by Higgins (36). For meta-analyses, *α* = 0.05 was chosen as the significance level. Sensitivity analyses were performed by removing individual studies to evaluate robustness for outcomes with considerable heterogeneity. For primary outcomes, predefined subgroup analyses were carried out based on the kind of disease. Egger’s test and funnel plots were used to evaluate publication bias. The trim and fill method was used to review and repair studies that were at high risk of bias. For studies that did not directly report the standard deviation (SD), recognized formulas were used to estimate SD (36–38), with detailed formulas provided in Supplementary Table 2.

## 3 Results

**Figure 2:**
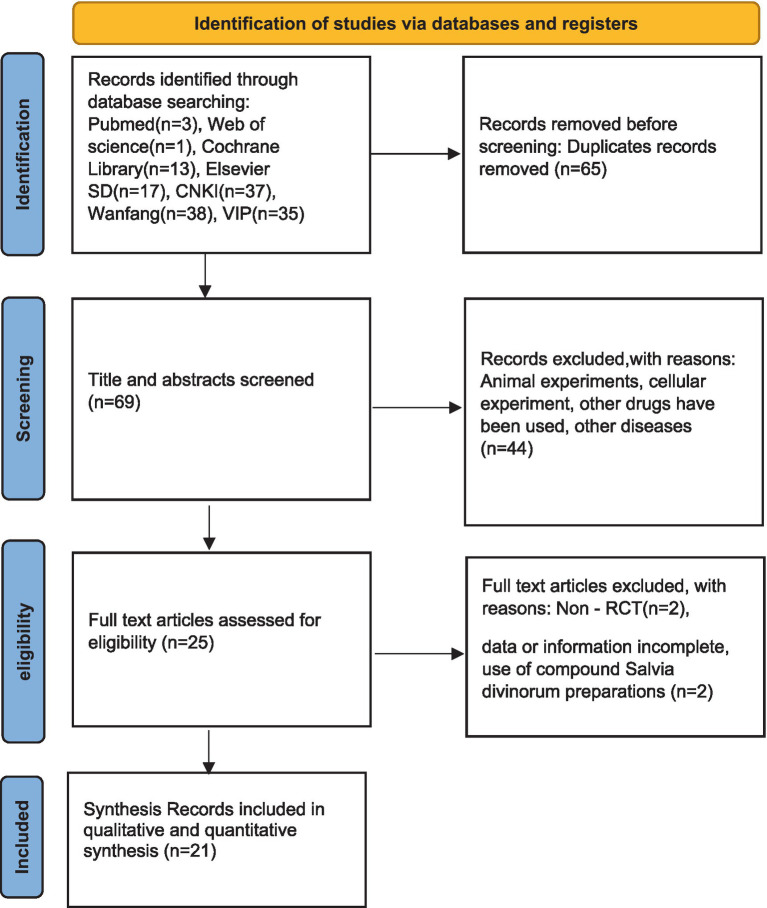
Flow plot.

After searching the databases, we identified 134 potentially eligible reports. We then reviewed the full text of 68 reports after removing duplicates and screening the titles and abstracts. In the end, 21 reports that satisfied every requirement for inclusion were added to the meta-analysis: Cai ZZ (2023), Li DS (2024), Liu RY (2024), Ren SC (2023), Wu XD (2014), Yu XY (2024), Zhang ZJ (2024), Wang Y (2025), Wang LH (2017), Gao C (2015), Zhang YF (2021), Zhou ZW (2022), Dong XJ (2023), Gong *F* (2024), Hou YY (2020), Shan JC (2016), Yan RQ (2013), Zhang L (2024), Qin H (2024), and Xu XQ (2023) (39–59). The detailed study selection process is summarized in Figure 2.

### 3.1 Study characteristics

A sample of 2084 individuals, 1,045 in the experimental (QKPC) group and 1,039 in the control group, which covers research conducted between 2013 and 2025. The length of treatment varied from5 to 28 days. AECOPD, AEA, or AB were the diagnoses used to classify the participants. Key baseline data also include age, gender, smoking history, and other relevant factors. The studies’ features are summed up in Table 1. The experimental group got QKPC in addition to normal care, which comprised oxygen therapy, antibiotics, and nebulized bronchodilators or corticosteroids. Details of concomitant medications are provided in Supplementary Table 3.

**Table 1:** Baseline data table.

Study author, year	Disease	Intervention measures		Sample sizeT/C	Therapy duration	Gender(male)T/C	Age T/C	Smoking history T/C	Outcome measures
Cai et al. (50)	AECOPD	Conventional western medicine +QKPC Granules	Conventional western medicine	40/40	7d	27/30	65.80 ± 5.31/63.68 ± 8.70	—	a,b,c,i
Li et al. (39)	AECOPD	Conventional western medicine +QKPC Granules	Conventional western medicine	40/40	14d	25/28	63.63 ± 6.94/64.23 ± 6.36	—	a,b,c,d,f,
Liu et al. (55)	AECOPD	Conventional western medicine +QKPC Granules	Conventional western medicine	50/50	14d	33/28	71.20 ± 8.83/74.93 ± 8.18	—	a,c,d,f,g,h
Ren et al. (56)	AECOPD	Conventional western medicine +QKPC Granules	Conventional western medicine+QKPC Granules placebo	28/28	14d	28/27	67.29 ± 7.37/69.54 ± 6.16	28/24^*^	b,f
Wu et al. (40)	AECOPD	Conventional western medicine +QKPC Granules	Conventional western medicine	80/80	7d	50/47	63.0 ± 12.5/61.7 ± 11.8	—	a,d,e,i
Yu et al. (54)	AECOPD	Conventional western medicine +QKPC Granules	Conventional western medicine	48/47	14d	32/27	64.8 ± 10.2/66.4 ± 9.5	24.52 ± 22.06/18.46 ± 23.36^#^	a,b,f,g,h,i
Zhang et al. (57)	AECOPD	Conventional western medicine +QKPC Granules	Conventional western medicine	50/50	14d	35/29	73.54 ± 10.05/75.76 ± 9.02	—	a,b,c,f,g,h
Wang (47)	AECOPD	Conventional western medicine +QKPC Granules	Conventional western medicine+Salbutamol Aerosol	41/41	14d	23/22	66.12 ± 2.34/66.51 ± 2.48	—	b,c,d,e,i
Wang (59)	asthma	Conventional western medicine +QKPC Granules	Conventional western medicine	32/32	7d	20/22	36.4 ± 6.21/35.8 ± 7.11	—	a
Gao (52)	asthma	QKPC Granules	Conventional western medicine	30/30	5d	17/16	7.01 ± 2.20/7.33 ± 2.31	—	a,b,d,e
Zhang et al. (49)	asthma	Conventional western medicine +QKPC Granules	Conventional western medicine	50/50	5d	25/26	6.89 ± 2.56/6.78 ± 2.49	—	a,b,j
Zhou et al. (46)	asthma	Conventional western medicine +QKPC Granules	Conventional western medicine	50/50	28d	32/21	45.46 ± 2.67/44.13 ± 3.45	—	a,b,d,k
Dong et al. (44)	asthma	Conventional western medicine +QKPC Granules	Conventional western medicine	30/30	5d	20/17	6.00 ± 2.46/6.27 ± 2.74	—	a,d,e,j
Gong et al. (43)	asthma	Conventional western medicine +QKPC Granules	Conventional western medicine	68/68	28d	35/37	9.79 ± 0.58/9.84 ± 0.62	—	a,b,e,j,k
Hou et al. (42)	cough	Conventional western medicine +Cupping therapy+QKPC Granules	Conventional western medicine+Cupping therapy	30/30	10d	12/13/15	44.87 ± 9.11/3.36 ± 1.84	—	a,l,n
Shan et al. (51)	cough	Conventional western medicine +QKPC Granules	Conventional western medicine	60/60	5-7d	-	3.18 ± 1.54/3.36 ± 1.84	—	a,l
Yan et al. (53)	cough	QKPC Granules	Conventional western medicine	50/50	5d	28/26	1–14	—	a
Zhang and Ji (41)	cough	Conventional western medicine +QKPC Granules	Conventional western medicine	48/45	7d	25/22	44.91 ± 6.69/45.61 ± 8.30	—	a,c,l,m
Yu et al. (48)	cough	Conventional western medicine +QKPC Granules	Conventional western medicine	80/80	7d	39/42	6.02 ± 2.01/5.68 ± 2.52	—	a,b,l,m,n
Qin et al. (45)	cough	Conventional western medicine +QKPC Granules	Conventional western medicine	90/88	7d	43/39	55.89 ± 13.79/53.66 ± 15.02	—	a,b,c,l
Xu et al. (58)	cough	Conventional western medicine +QKPC Granules	Conventional western medicine	50/50	14d	27/22	43.6 ± 8.5/44.2 ± 9.1	—	a

a: Efficacy; b: Safety; c: CRP; d: FEV1%; e: FVC; f: CAT; g:6WMT; h:mMRC; i: PaO₂; j: PEF; k: IgE; l: Cough disappearance time; m: TNF-a; n: IL-1β; *number of patients with a history of smoking; #Smoking index.

### 3.2 Risk of bias assessment

**Figure 3:**
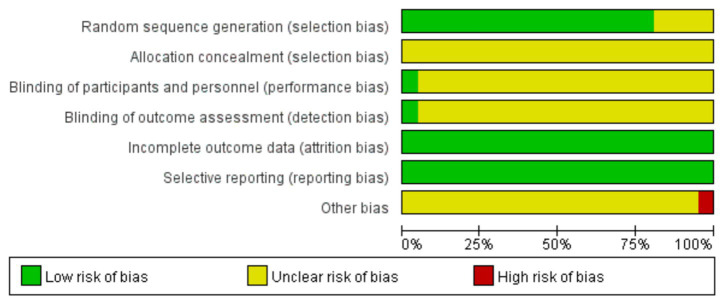
Risk of bias graph.

The Cochrane Risk of Bias Tool was used to perform a comprehensive quality assessment of the literature. Based on the 21 RCTs, 17 studies were classified as “low risk” for this domain, and sequences were generated using random number tables; Four studies were rated as “uncertain risk” because they did not describe the randomization procedure; There was “uncertain risk” for all studies since allocation concealment was not mentioned in any study; Ren et al. were rated “low risk” for deviating from intended interventions and outcome assessment since they explicitly disclosed double-blinding; all other trials were rated “uncertain risk” because they did not report blinding; Every study provided comprehensive outcome data that showed no signs of attrition bias (“low risk”); all were categorized as “low risk” since they reported all prespecified outcome measures without selective omission. One study (Hou YY, 2020) combined QKPC with cupping therapy, introducing a potential confounding intervention; this study was rated “high risk” for other bias. Detailed assessments are presented in Figure 3.

### 3.3 Primary outcomes

**Figure 4:**
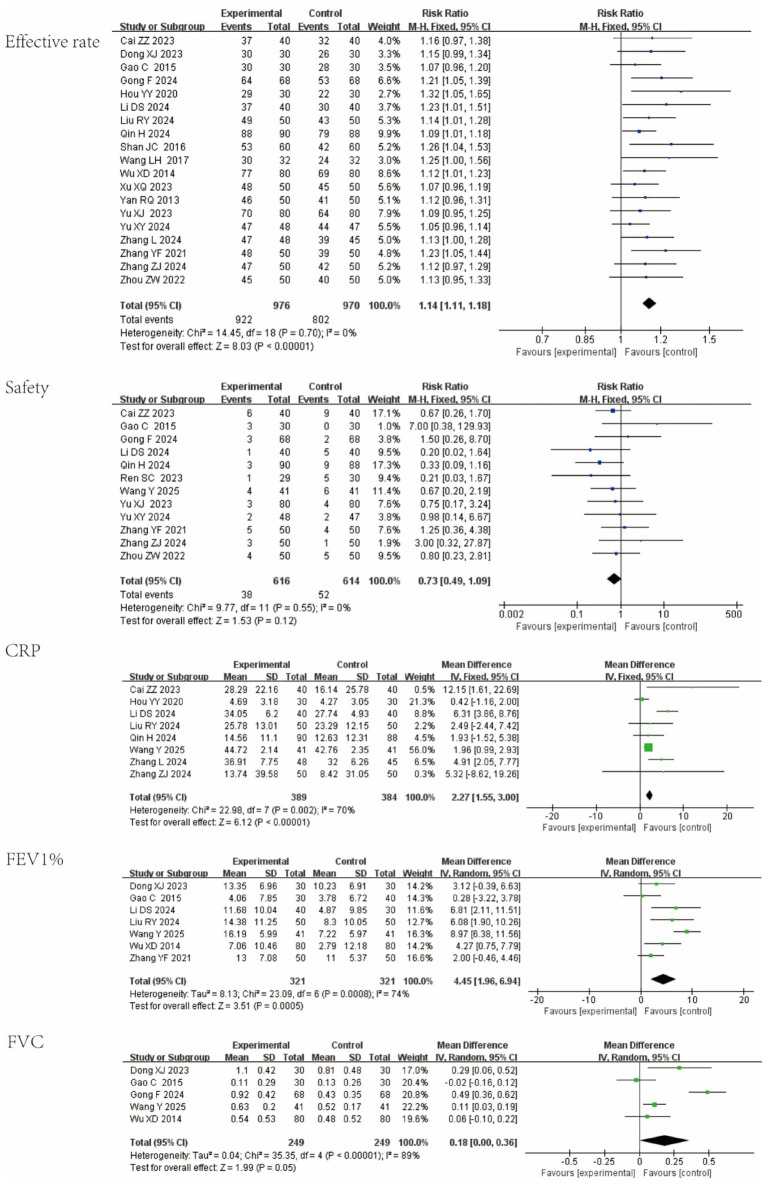
Forest plot of primary outcomes for Qingke Pingchuan granules.

Nineteen studies reported overall response rate; 12 reported AEs; 8 reported CRP; 7 reported FEV₁% predicted; and 5 reported FVC. Compared with control interventions, QKPC improved clinical efficacy and reduced systemic inflammation, with favorable safety. Pooled estimates are as follows: Effective rate: RR = 1.14 (95% CI [1.11, 1.18]; I^2^ = 0%; *p* < 0.00001); CRP: MD = 2.77 (95% CI [1.55, 3.00]; I^2^ = 70%; *p* < 0.0001); FEV₁%: MD = 4.46 (95% CI [3.23, 5.69]; I^2^ = 74%; *p* = 0.0005); FVC: MD = 0.18 (95% CI [0.00, 0.36]; I^2^ = 89%; *p* = 0.05). Analysis of adverse events revealed no statistically significant difference in the overall incidence of adverse reactions between the Qingke Pingchuan Granules plus conventional therapy group and the conventional therapy alone group (RR = 0.73 [95% CI 0.49, 1.09]; I^2^ = 0%; *p* = 0.12). All reported adverse reactions were mild, primarily including gastrointestinal discomfort, rash, and transient dizziness. No serious adverse reactions occurred in either group, and no liver or kidney function impairment was observed in any study, further confirming the good safety profile of QKPC as an adjunctive therapy. Forest plots for all primary outcomes are shown in Figure 4.

**Figure 5:**
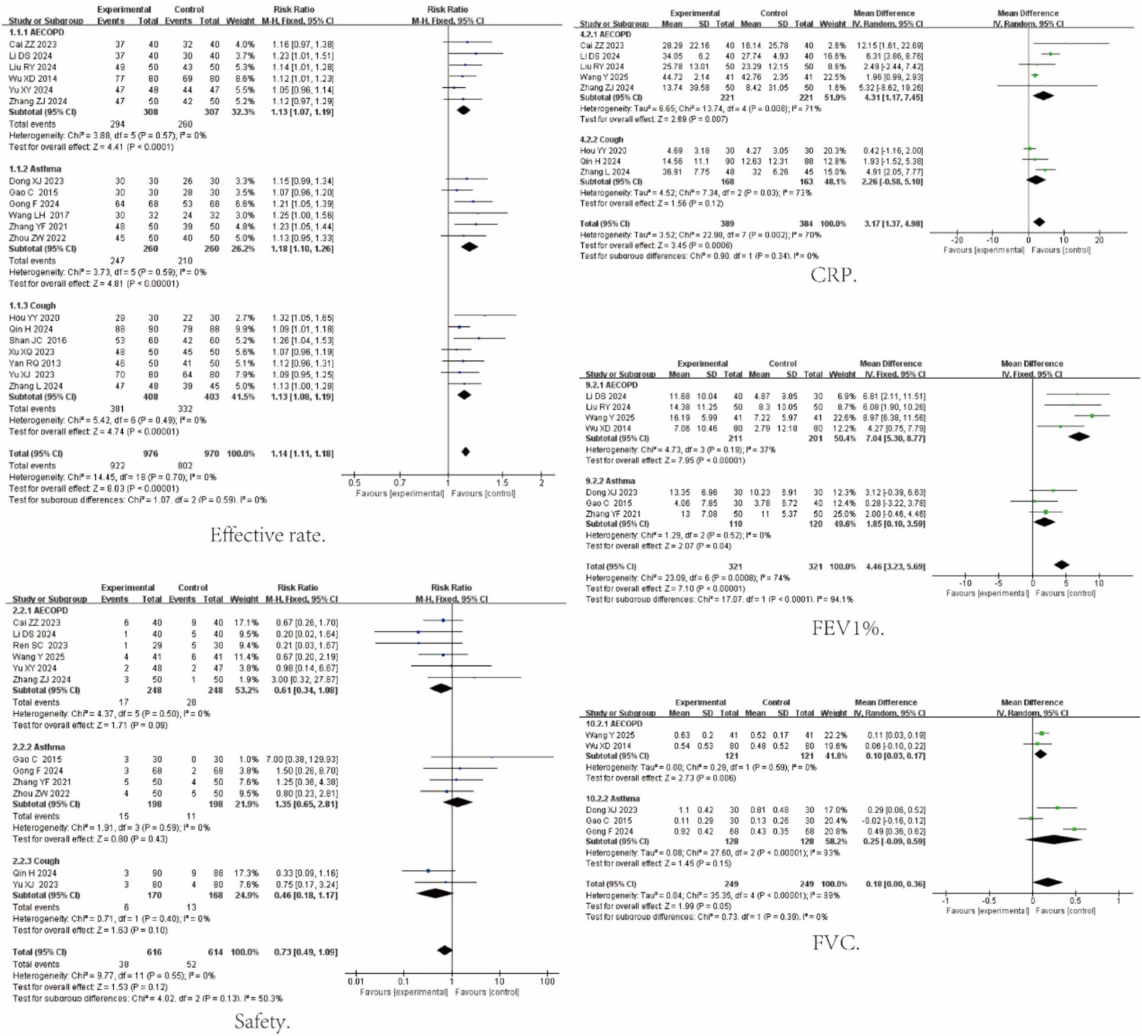
Disease-specific subgroup analysis.

To address potential clinical heterogeneity arising from the pooled analysis of three diseases with distinct pathophysiological characteristics, we further performed disease-specific subgroup analyses focusing on key outcome measures. Results showed no significant differences in response rate and safety indicators across the three diseases, whereas notable subgroup-specific variations were observed in CRP, FEV₁% and FVC. In AECOPD patients, significant improvements were achieved in CRP (MD = 4.31, 95%CI [1.17, 7.45]; I^2^ = 71%; *p* = 0.007), FEV₁% (MD = 7.04, 95% CI [5.30, 8.77]; I^2^ = 37%; *p* < 0.0001), and FVC (MD = 0.10, 95% CI [0.03, 0.17]; I^2^ = 0%; *p* = 0.006). This confirms the stable therapeutic efficacy of QKPC in this subgroup. In the AEA subgroup, although FEV₁% showed a significant improvement (MD = 1.85, 95% CI [1.17, 7.45]; I^2^ = 0%; *p* = 0.04), the change in FVC (MD = 0.25, 95% CI [−0.09, 0.59]; I^2^ = 93%; *p* = 0.05) was not statistically significant. For the AB subgroup, the improvement in CRP (MD = 2.26, 95% CI [−0.58, 5.10]; I^2^ = 73%; *p* = 0.12) failed to reach statistical significance. These findings are elaborated in Figure 5.

QKPC exhibited consistent therapeutic effects in AECOPD, AEA, and AB, validating its broad anti-inflammatory properties and ability to regulate symptoms. Notably, only the AECOPD subgroup demonstrated stable efficacy across CRP, FEV₁%, and FVC, while AEA and AB subgroups did not achieve expected improvements in inflammation and lung function, warranting further in-depth investigations. Additionally, subgroup analysis revealed a significant difference in the magnitude of FEV₁% improvement between asthma and COPD patients after treatment. This phenomenon is most likely attributed to the inherent differences in airway pathophysiological characteristics between the two diseases: airflow limitation in asthma is characterized by variability and reversibility, whereas that in COPD is predominantly irreversible. These differences may account for the varying degrees of FEV₁% improvement observed. Therefore, the impact of QKPC on the aforementioned two lung function indicators in AEA patients requires more comprehensive and in-depth studies for validation.

All studies included in the present meta-analysis were classified as the phlegm-heat syndrome subtype; however, the diagnostic criteria for this TCM syndrome lacked uniformity across the included trials. We therefore conducted subgroup analyses of effective rate of core and FEV1% stratified by the adopted TCM diagnostic criteria. Specifically, 12 studies conformed to the standard criteria specified in the Guiding Principles for Clinical Research on New Chinese Medicinal Products and the Clinical Practice Guidelines for TCM in the Management of Chronic Obstructive Pulmonary Disease (33, 60), while 9 studies applied other diagnostic criteria. No statistically significant differences were identified between these two subgroups for primary outcome measures, which suggests that variable diagnostic criteria for the phlegm-heat TCM syndrome are not a key source of heterogeneity in the current analysis. Detailed findings are shown in Supplementary Figure 2.

### 3.4 AECOPD-specific outcomes

**Figure 6:**
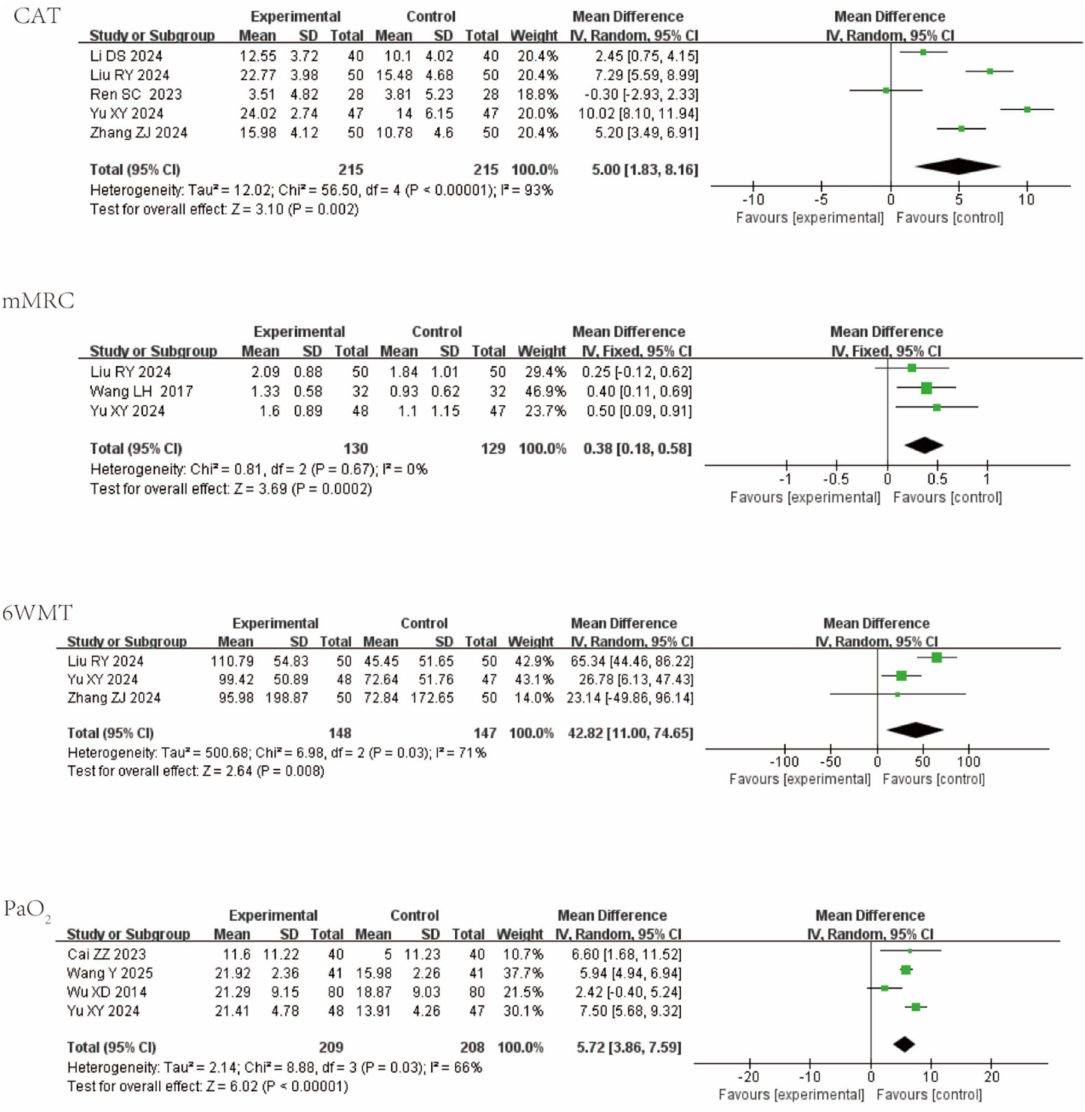
Forest plot of AECOPD-relevant outcomes.

Five studies reported the CAT score; three reported the mMRC dyspnea scale; three reported the 6MWT; and four reported PaO₂. The QKPC group outp CAT score: MD = 5.00 (95% CI [1.83, 8.61]; I^2^ = 93%; *p* = 0.002); I^2^ = 93%; *p* = 0.002; mMRC: MD = 0.37 (95% CI [0.17, 0.58]; I^2^ = 0%; *p* = 0.0004); 6MWT: MD = 42.82 (95% CI [11.00, 74.65]; I^2^ = 71%; *p* = 0.008); PaO₂: MD = 5.72 (95% CI [3.86, 7.59]; I^2^ = 66%; *p* < 0.0001). Forest plots are shown in Figure 6.

### 3.5 Asthma-specific outcomes

**Figure 7:**
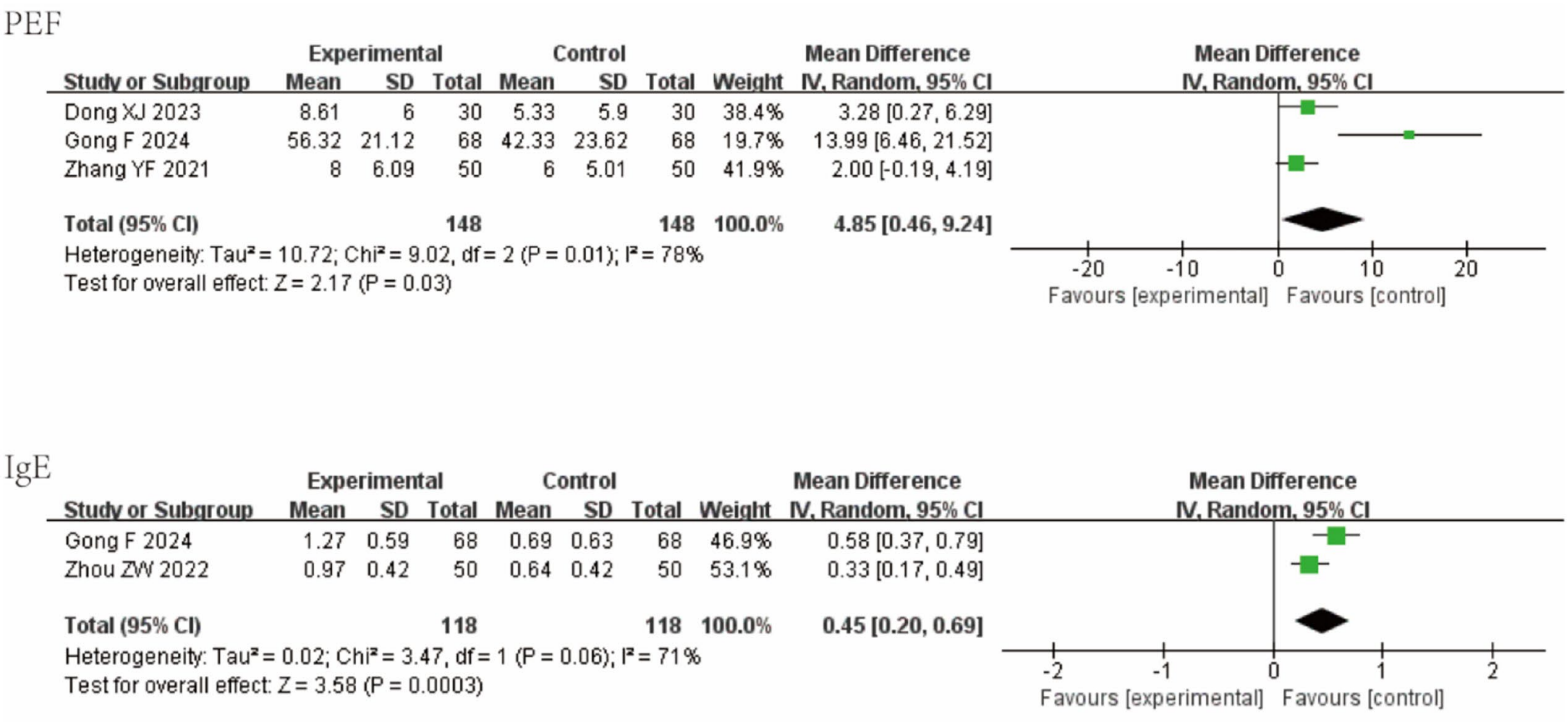
Forest plot of AEA-relevant outcomes.

Three studies reported PEF; two studies reported IgE levels. In acute asthma exacerbation, QKPC significantly improved both functional and immunological parameters: PEF: MD = 4.85 (95% CI [0.46, 9.24]; I^2^ = 78%; *p* = 0.03); IgE: MD = 0.45 (95% CI [0.20, 0.69]; I^2^ = 71%; *p* = 0.0003). Forest plots are shown in Figure 7.

### 3.6 Acute bronchitis–specific outcomes

**Figure 8:**
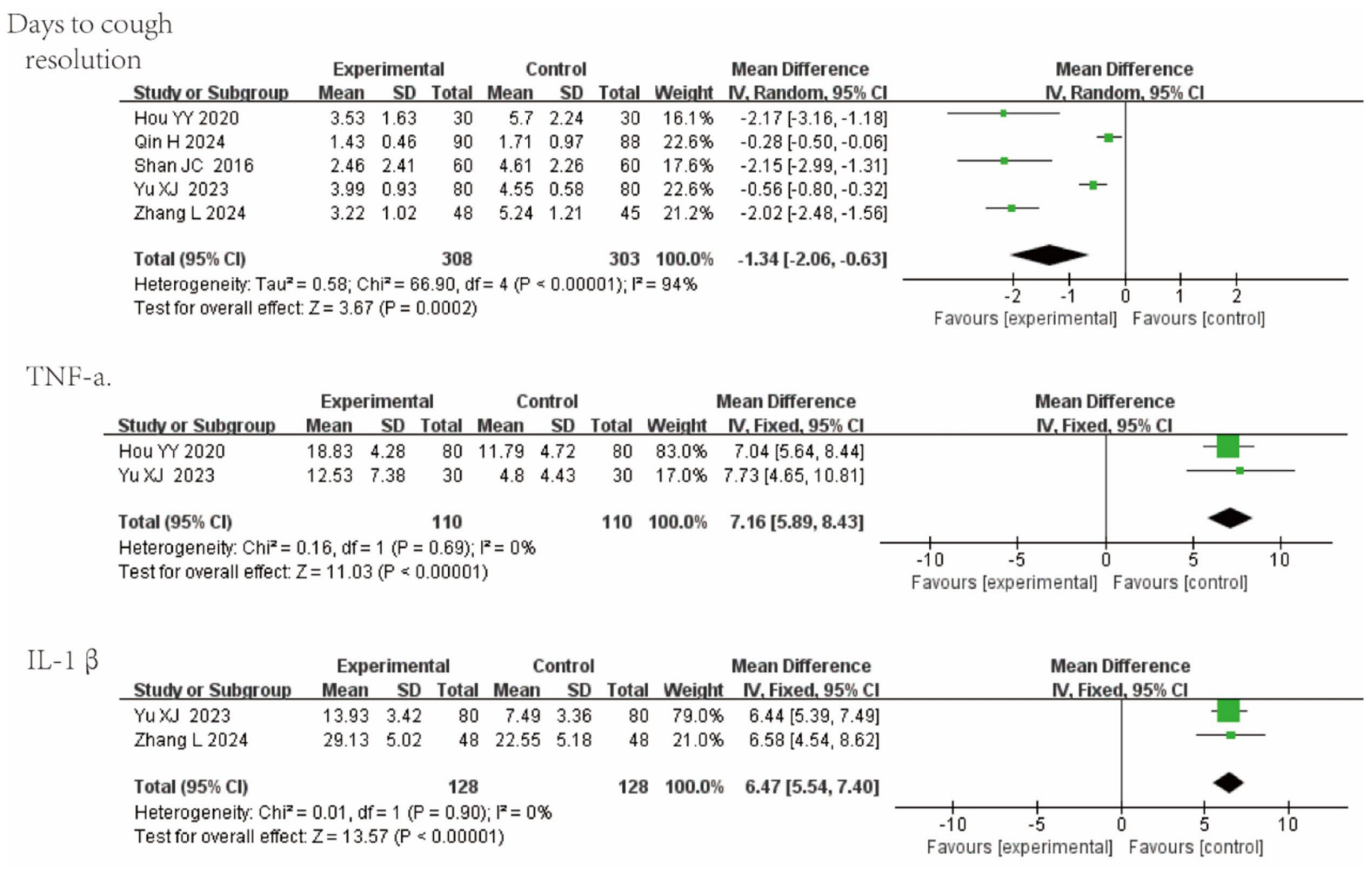
Forest plot of AB-relevant outcomes.

Five studies reported days to cough resolution; two reported tumor necrosis factor-alpha (TNF-*α*); and two reported IL-1β. QKPC significantly accelerated cough resolution and reduced key pro-inflammatory cytokines in acute infectious cough: Days to cough resolution: MD = −1.34 (95% CI [−2.06, −0.63]; I^2^ = 94%; *p* = 0.0002); TNF-α: MD = 7.16 (95% CI [5.89, 8.43]; I^2^ = 0%; *p* < 0.00001); IL-1β: MD = 6.47 (95% CI [5.54, 7.40]; I^2^ = 0%; *p* < 0.00001). Forest plots are shown in Figure 8.

### 3.7 Sensitivity analysis and publication bias

We conducted sensitivity analysis via Stata software. The results showed that after removing the study by Wu, the heterogeneity of PaO₂ was significantly reduced (I^2^ decreased from 66 to 8%), indicating that this study was the primary source of heterogeneity for this outcome measure. For other outcome measures, the pooled effect size showed no significant changes after excluding any individual study, demonstrating the robustness of the overall analysis results. Detailed results are presented in Supplementary Figure 3.

**Figure 9:**
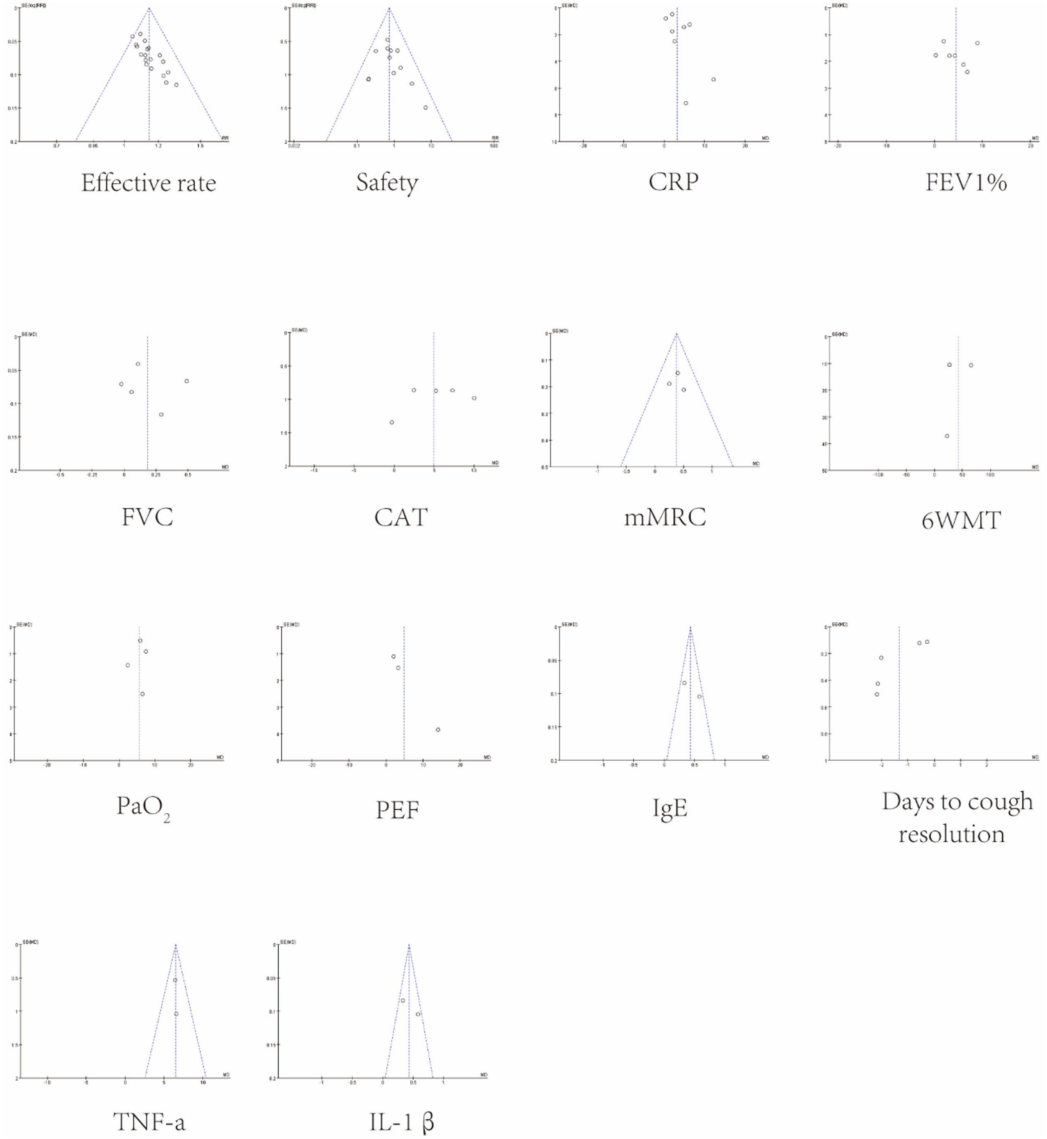
Funnel plots.

**Figure 10:**
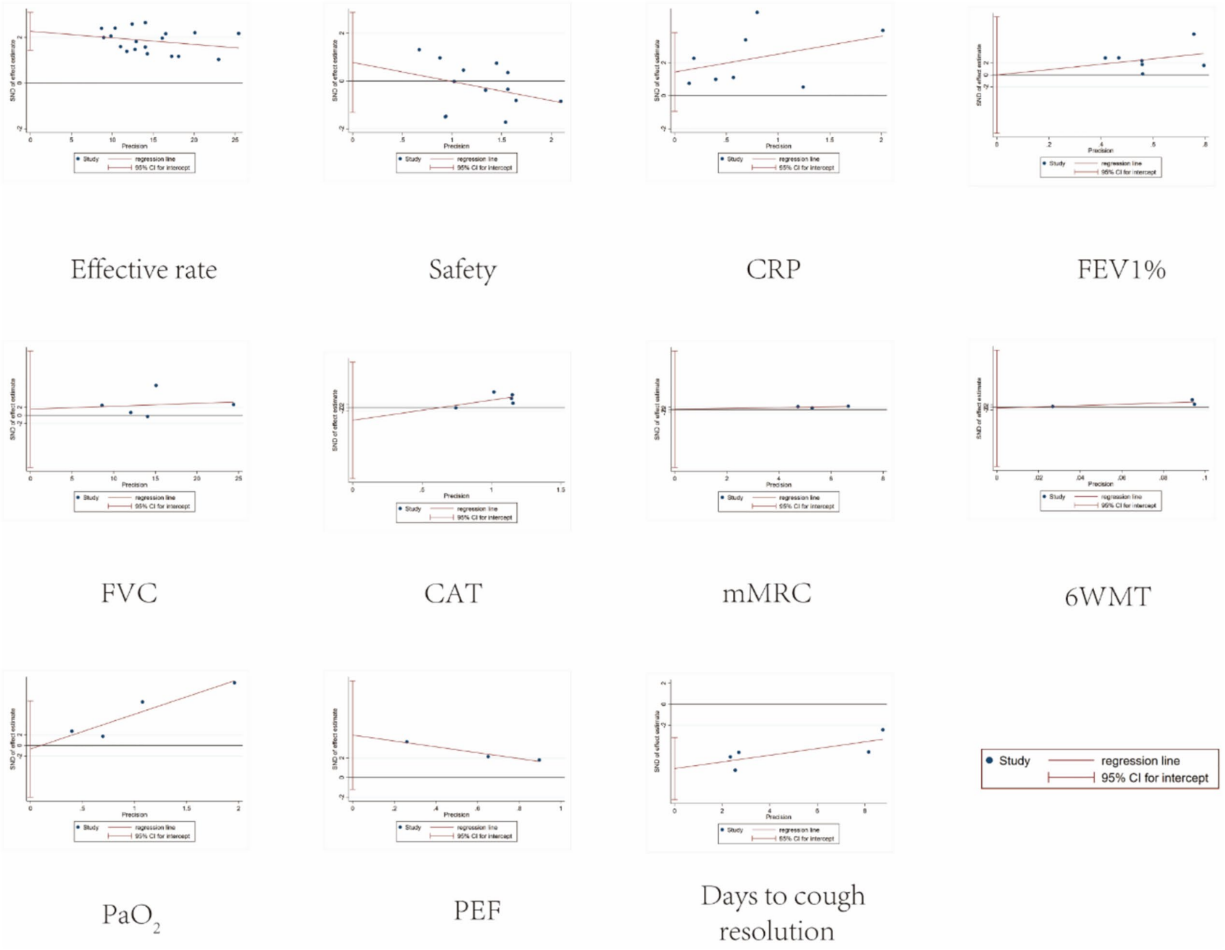
Egger plots.

Funnel plots showed no obvious asymmetry across outcomes (see Figure 9). Egger’s regression test revealed publication bias for the Effective rate (*p* = 0.000) and days to cough resolution (*p* = 0.007), and the remaining data showed no publication bias (*p* > 0.05). Detailed results are presented in Figure 10. To evaluate the possible influence of this bias on the combined effect size, the trim-and-fill method was implemented. The analysis of the Effective rate indicated that seven studies may be missing; after repair, the adjusted overall number of studies rose to 26. The overall effect size was recalculated using the fixed effects model, and the result was RR = 1.09, 95% CI [1.067, 1.126]; *p* < 0.001. Days to cough resolution indicated no missing studies. After repair, the result was MD = 0.264 (95% CI [0.135, 0.518]; *p* < 0.001), and the effect value remained statistically significant, indicating that the current study’s findings were still reliable. Detailed results are presented in Supplementary Figures 4, 5.

## 4 Discussion

Acute airway inflammatory diseases, including AECOPD, AEA, and AB, present a serious challenge for public health systems and place a significant burden on patients’ daily lives (61). However, the worsening process is consistently characterized by oxidative stress and inflammatory activity (62, 63). The development of these acute airway conditions involves multiple factors, that is to say, things like environmental exposures, infections that trigger responses, and immune responses that aren’t properly regulated (64, 65). Clinically, it is important to note that these three disease types show quite similar symptoms and treatment approaches. Regarding symptoms, they typically feature things like reduced lung function, higher levels of inflammatory markers throughout the body and in airways, plus increased coughing, more phlegm production, and wheezing sounds (66, 67). Based on the theory of TCM, the acute worsening stages of AECOPD, AEA, and AB mostly fall under the “phlegm-heat obstructing the lung” pattern, with the core issue being internal buildup of phlegm-heat along with the lung’s qi not spreading and descending properly.

In terms of treatment, they are commonly managed with inhaled anti-inflammatory drugs, mucolytic agents, and antitussive agents (68, 69). Although conventional therapies play a critical role in acute symptom control, their long-term or repeated use is limited by adverse effects and risks of antibiotic overuse (24, 70). TCM can provide targeted therapy against the core pathogenesis of “phlegm-heat” in these diseases. The association between the “phlegm-heat obstructing the lung” syndrome and inflammation, as well as oxidative stress, has been confirmed by multiple studies (71, 72). Relevant studies have demonstrated that TCM can intervene in multiple pathological processes of airway diseases, such as oxidative stress, inflammatory response, and ferroptosis, thus holding significant value as an adjuvant clinical therapy (73–75).

QKPC inherits the formulation of Maxing Shigan Tang, as recorded in Zhang Zhongjing’s Shanghan Lun over 1,800 years ago (76). This traditional approach from Chinese medicine shows use in the treatment of conditions relating to breathing problems across multiple centuries. Clinically, it emphasizes the rational compatibility of therapeutic principles such as clearing heat, dispersing lung qi, and relieving asthma, which is highly consistent with the targeted treatment of phlegm-heat-type airway diseases (30). Preclinical evidence further supports the therapeutic potential of QKPC. Mengjiao Xu examined a model using cigarette smoke and LPS to produce the condition and showed that QKPC reduces injury to the airway covering by affecting the function of structures that neutrophils release to the outside (30). Yuanyuan Wu reported that QKPC decreases the disruption of particular cells in the airway, improves the process of repair in the covering, and reduces injury to the lung (31). Linqing He showed that QKPC works to prevent activity in the pathway involving TLR7/NF-κB, and this reduces replication of virus and production of factors that produce inflammation in models examining viral infection of the airway (77). To date, 108 bioactive components have been identified in QKPC, among which quercetin, luteolin, and gallic acid are recognized as the core bioactive components. The broad-spectrum anti-inflammatory and symptom-regulating activities of QKPC are likely attributable to these three compounds (30). Relevant studies have demonstrated that quercetin can target mast cells, inhibit the activation of the NF-κB and MAPK signaling pathways, and reduce the overexpression of pro-inflammatory cytokines such as TNF-*α* and IL-1*β* (78), which is consistent with the marked reduction in TNF-α and IL-1β levels observed in the AB subgroup of the present study. As a flavonoid compound, luteolin exerts a favorable protective effect on airway epithelium (79). It can alleviate airway inflammation by regulating macrophage polarization and attenuate airway remodeling via its action on β-catenin in airway epithelial cells (79, 80), thereby providing a mechanistic basis for QKPC to improve lung function and relieve airway hyperresponsiveness in AECOPD and AEA patients. Oxidative stress imbalance and lipid peroxidation injury are key links in the pathological process of AECOPD. Gallic acid enhances antioxidant capacity by activating the Nrf2/HO-1 pathway and reduces the level of malondialdehyde (MDA), a lipid peroxidation product, thus effectively mitigating airway injury in mice exposed to LPS (81). Based on the airway protective activity of gallic acid, it is speculated that it may be one of the key bioactive components of QKPC responsible for improving clinical outcomes in AECOPD patients. Collectively, these findings highlight the pleiotropic mechanism of QKPC, including anti-inflammation, epithelial protection, immunomodulation, and antiviral activity that underpin its broad applicability in acute airway diseases.

This review systematically assessed the efficacy and safety of QKPC in three acute airway diseases. QKPC exhibited beneficial effects on the total effective rate, adverse events, lung function, and CRP levels in patients with acute airway diseases. Notably, subgroup analysis showed that the AECOPD subgroup achieved stable therapeutic effects on both inflammation and lung function, while the AEA and AB subgroups failed to reach expected improvements in these two aspects. At the same time, subgroup analysis of FEV1% revealed a significant difference between the AEA and AECOPD groups.s. This discrepancy may be attributed to the reversible airway obstruction in asthma, whereas COPD is characterized by irreversible airway impairment. In addition to disease-based subgroup analyses, we further conducted subgroup analyses on the diagnostic criteria for traditional Chinese medicine syndrome differentiation and classification. Results of these analyses showed consistent efficacy of QKPC for this syndrome under both sets of criteria, which further substantiates the stable targeted therapeutic effect of QKPC on the “phlegm-heat obstructing the lung syndrome” in acute airway diseases. For AECOPD, QKPC was reported to enhance 6MWT distance, lower mMRC and CAT scores, and improve PaO₂. Notably, utilizing sensitivity analysis, the variability of PaO₂ outcomes was significantly reduced, and the aggregated results stayed consistent after removing one study (40). QKPC has been demonstrated to lower IgE levels and enhance PEF for AEA. Nevertheless, there were only a few included studies, and more RCTs should be conducted in the future. QKPC reduced serum levels of TNF-*α* and IL-1β and limited days of cough resolution for AB. To sum up, QKPC greatly improves patients’ quality of life, lowers inflammatory factor levels, and relieves the symptoms of acute airway illnesses.

This review is the first systematic Meta-analysis evaluating QKPC for the treatment of acute airway diseases, with unique advantages. Firstly, it focuses on diseases sharing acute airway inflammation as the common core mechanism, all of which fall into the category of phlegm-heat syndrome and exhibit high similarity in clinical symptoms and treatment approaches. This study included 21 RCTs, systematically integrated efficacy and safety of three types of acute airway diseases, and comprehensively analyzed the advantages of QKPC in treating phlegm-heat-type airway diseases. Secondly, for outcome measures, a combination of overall analysis and disease-specific analysis was adopted to fully reveal the therapeutic effects of QKPC on the three diseases through multi-dimensional evaluation. In addition, this study employed sensitivity analysis, funnel plot visualization, Egger’s linear regression test, and the trim-and-fill method to correct publication bias, verifying the robustness of the results from multiple aspects. Results show that QKPC exhibits certain advantages in improving clinical symptoms, enhancing lung function, and inhibiting inflammatory responses. This study provides research avenues for future high-quality RCTs and provides rigorous scientific support for the evidence-based clinical application of QKPC.

Despite these advantages, there are a few drawbacks that should be carefully considered: First, a number of outcomes showed significant heterogeneity. Fundamental pathophysiological variations are one possible source; second, treatment length diversity among trials may have an impact on the degree and timing of functional gains. Additionally, all of the included RCTs had small sample sizes, were single-center studies carried out in China, and had little representation in English-language databases. Third, there is still little evidence for a number of important consequences. For example, just two studies each reported IL-1β and IgE, which limited the pooled estimates’ statistical power and generalizability. Fourthly, a subset of the included studies had inadequate reporting of confounding factors such as smoking history and details of conventional treatment. The types of conventional treatment and specific intervention measures were not fully specified, precluding further in-depth analysis. However, sensitivity analysis indicated that these factors did not affect the robustness of the primary outcomes, supporting the reliability of the conclusions.

We suggest the following suggestions for further research in light of these limitations: First, high-quality, multicenter, large-sample, long-term follow-up RCTs are desperately needed, with more focus on research design uniformity and transparency. Secondly, expand the study of translational mechanisms. Provide a pharmacological foundation for precision dosing by combining pharmacokinetic analyses with clinically well-phenotyped cohorts to clarify how the active ingredients of Qingke Pingchuan Granules work in airway disorders. Lastly, to expand the scope of the medication’s therapeutic use, concentrate on its safety and effectiveness in patients with airway comorbidities.

In conclusion, through systematic review and meta-analysis, this article confirmed that Qingke Pingchuan Granules in conjunction with conventional Western medicine treatment exerts a significant positive effect on the clinical efficacy, lung function, inflammatory status, and quality-of-life related scores in patients with AECOPD, AEA, and AB. It does not increase the risk of adverse reactions and thus serves as a safe and effective adjuvant therapeutic option for these three acute airway inflammatory diseases. Despite certain limitations, the findings of this study provide important evidence-based references for the clinical application of Qingke Pingchuan Granules, and also point out the direction for high-quality subsequent research on TCM in the treatment of acute airway diseases.

## 5 Conclusion

Acute airway inflammatory diseases are a category of phlegm-heat-type diseases. This study offers evidence that QKPC is a successful adjuvant therapeutic regimen for such diseases, even though heterogeneity within disease subtypes is still an issue. Among the disease types evaluated, the evidence for AECOPD is the most favorable therapeutic effect. The combined regimen can significantly increase the clinical Effective rate, FEV₁%, FVC, and PaO₂ levels in AECOPD patients; it can also reduce CAT, mMRC scores, as well as the levels of inflammatory factors, while improving exercise capacity indicators, including the 6MWT. For patients with AEA and AB, this regimen can also alleviate core clinical symptoms and ameliorate inflammatory statusNevertheless, further well-designed studies are warranted to validate these findings. Based on the core results of the present study, QKPC is recommended for preferential clinical application in AECOPD patients presenting with the phlegm-heat obstructing the lung syndrome. Combination with conventional Western medical treatment is advisable. For patients with weak spleen and stomach function, administration after meals is recommended. QKPC is contraindicated in patients allergic to any of its ingredients, those with phlegm-cold obstructing the lung syndrome, and individuals with severe hepatic or renal insufficiency. No dosage adjustment is required for elderly patients; however, regular monitoring of hepatic and renal function should be performed. These findings indicate that the rational application of Qingke Pingchuan Granules can target and ameliorate acute airway inflammation, thus serving as a safe and effective clinical therapeutic intervention.

## Data Availability

The datasets supporting the conclusions of this article are included within the article.
