# Supplementary material for: Qingke Pingchuan granules as adjuvant therapy for acute exacerbation of chronic obstructive pulmonary disease, acute exacerbation asthma, and acute bronchitis: a systematic review and meta-analysis
Source: Front Med (Lausanne). 2026 Mar 17;13:1772299. doi: 10.3389/fmed.2026.1772299 (PMC13036208; doi:10.3389/fmed.2026.1772299)
Supplement: Supplementary file 1 [file Table_1.DOCX]

| Study author, year | disease | Intervention measures | | Sample sizeT/C | Therapy duration | Gender（male）T/C | Age T/C | Smoking history T/C | Outcome Measures |
| --- | --- | --- | --- | --- | --- | --- | --- | --- | --- |
| Cai ZZ 2023 | AECOPD | Conventional western medicine +QKPC Granules | Conventional western medicine | 40/40 | 7d | 27/30 | 65.80±5.31/63.68±8.70 | — | a,b,c,i |
| Li DS 2024 | AECOPD | Conventional western medicine +QKPC Granules | Conventional western medicine | 40/40 | 14d | 25/28 | 63.63±6.94/64.23±6.36 | — | a,b,c,d,f, |
| Liu RY 2024 | AECOPD | Conventional western medicine +QKPC Granules | Conventional western medicine | 50/50 | 14d | 33/28 | 71.20±8.83/74.93±8.18 | — | a,c,d,f,g,h |
| Ren SC 2023 | AECOPD | Conventional western medicine +QKPC Granules | Conventional western medicine+QKPC Granules placebo | 28/28 | 14d | 28/27 | 67.29±7.37/69.54±6.16 | 28/24^*^ | b,f |
| Wu XD 2014 | AECOPD | Conventional western medicine +QKPC Granules | Conventional western medicine | 80/80 | 7d | 50/47 | 63.0±12.5/61.7±11.8 | — | a,d,e,i |
| Yu XY 2024 | AECOPD | Conventional western medicine +QKPC Granules | Conventional western medicine | 48/47 | 14d | 32/27 | 64.8±10.2/66.4±9.5 | — | a,b,f,g,h,i |
| Zhang ZJ 2024 | AECOPD | Conventional western medicine +QKPC Granules | Conventional western medicine | 50/50 | 14d | 35/29 | 73.54±10.05/75.76±9.02 | 24.52±22.06/18.46±23.36^#^ | a,b,c,f,g,h |
| Wang Y 2025 | AECOPD | Conventional western medicine +QKPC Granules | Conventional western medicine+Salbutamol Aerosol | 41/41 | 14d | 23/22 | 66.12±2.34/66.51±2.48 | — | b,c,d,e,i |
| Wang LH 2017 | Asthma | Conventional western medicine +QKPC Granules | Conventional western medicine | 32/32 | 7d | 20/22 | 36.4±6.21/35.8±7.11 | — | a |
| Gao C 2015 | Asthma | QKPC Granules | Conventional western medicine | 30/30 | 5d | 17/16 | 7.01±2.20/7.33±2.31 | — | a,b,d,e |
| Zhang YF 2021 | Asthma | Conventional western medicine +QKPC Granules | Conventional western medicine | 50/50 | 5d | 25/26 | 6.89±2.56/6.78±2.49 | — | a,b,j |
| Zhou ZW 2022 | Asthma | Conventional western medicine +QKPC Granules | Conventional western medicine | 50/50 | 28d | 32/21 | 45.46±2.67/44.13±3.45 | — | a,b,d,k |
| Dong XJ 2023 | Asthma | Conventional western medicine +QKPC Granules | Conventional western medicine | 30/30 | 5d | 20/17 | 6.00±2.46/6.27±2.74 | — | a,d,e,j |
| Gong F 2024 | Asthma | Conventional western medicine +QKPC Granules | Conventional western medicine | 68/68 | 28d | 35/37 | 9.79±0.58/9.84±0.62 | — | a,b,e,j,k |
| Hou YY 2020 | Cough | Conventional western medicine +Cupping therapy+QKPC Granules | Conventional western medicine+Cupping therapy | 30/30 | 10d | 12/13/15 | 44.87±9.11/3.36±1.84 | — | a,l,n |
| Shan JC 2016 | Cough | Conventional western medicine +QKPC Granules | Conventional western medicine | 60/60 | 5-7d | - | 3.18±1.54/3.36±1.84 | — | a,l |
| Yan RQ 2013 | Cough | QKPC Granules | Conventional western medicine | 50/50 | 5d | 28/26 | 1-14 | — | a |
| Zhang L 2024 | Cough | Conventional western medicine +QKPC Granules | Conventional western medicine | 48/45 | 7d | 25/22 | 44.91±6.69/45.61±8.30 | — | a,c,l,m |
| Yu XJ 2023 | Cough | Conventional western medicine +QKPC Granules | Conventional western medicine | 80/80 | 7d | 39/42 | 6.02±2.01/5.68±2.52 | — | a,b,l,m,n |
| Qin H 2024 | Cough | Conventional western medicine +QKPC Granules | Conventional western medicine | 90/88 | 7d | 43/39 | 55.89±13.79/53.66±15.02 | — | a,b,c,l |
| Xu XQ 2023 | Cough | Conventional western medicine +QKPC Granules | Conventional western medicine | 50/50 | 14d | 27/22 | 43.6±8.5/44.2±9.1 | — | a |

**Table1:**Baseline data table: a:Efficacy; b:Safety; c:CRP; d:FEV1%; e:FVC; f:CAT; g:6WMT; h:mMRC; i:PaO₂; j:PEF; k:IgE; l:Cough disappearance time; m:TNF-a; n:IL-1β; *:number of patients with a history of smoking ; #: Smoking index
