# Supplementary material for: Qingke Pingchuan granules as adjuvant therapy for acute exacerbation of chronic obstructive pulmonary disease, acute exacerbation asthma, and acute bronchitis: a systematic review and meta-analysis
Source: Front Med (Lausanne). 2026 Mar 17;13:1772299. doi: 10.3389/fmed.2026.1772299 (PMC13036208; doi:10.3389/fmed.2026.1772299)
Supplement: Supplementary file 2 [file Data_Sheet_1.pdf]

supplementary materials

Supplementary table1.search strategy

PubMed

((Qingke Pingchuan) OR (Qingke Pingchuan Granule)) AND (((((((((((COPD) OR (Chronic Obstructive Pulmonary Disease, Acute Exacerbation)) OR (Asthma)) OR (Bronchial Asthma, Acute Exacerbation)) OR (Cough)) OR (Cough, Infectious, Acute)) OR (Pulmonary Disease, Chronic Obstructive[MeSH Terms])) OR (Chronic Obstructive Pulmonary Disease, Acute[MeSH Terms])) OR (Asthma-Chronic Obstructive Pulmonary Disease Overlap Syndrome[MeSH Terms])) OR (Asthma[MeSH Terms])) OR (Cough[MeSH Terms])) OR (Chronic Cough[MeSH Terms])) Sort by: Most Recent

(("Qingke"[All Fields] AND "Pingchuan"[All Fields]) OR ("Qingke"[All Fields] AND "Pingchuan"[All Fields] AND ("cytoplasmic granules"[MeSH Terms] OR ("cytoplasmic"[All Fields] AND "granules"[All Fields]) OR "cytoplasmic granules"[All Fields] OR "granule"[All Fields] OR "granulate"[All Fields] OR "granulated"[All Fields] OR "granulates"[All Fields] OR "granulating"[All Fields] OR "granulation"[All Fields] OR "granulations"[All Fields] OR "granulative"[All Fields] OR "granulator"[All Fields] OR "granulators"[All Fields] OR "granule s"[All Fields] OR "granules"[All Fields] OR "granulous"[All Fields]))) AND ("pulmonary disease, chronic obstructive"[MeSH Terms] OR ("pulmonary"[All Fields] AND "disease"[All Fields] AND "chronic"[All Fields] AND "obstructive"[All Fields]) OR "chronic obstructive pulmonary disease"[All Fields] OR "copd"[All Fields] OR (("pulmonary disease, chronic obstructive"[MeSH Terms] OR ("pulmonary"[All Fields] AND "disease"[All Fields] AND "chronic"[All Fields] AND "obstructive"[All Fields]) OR "chronic obstructive pulmonary disease"[All Fields] OR ("chronic"[All Fields] AND "obstructive"[All Fields] AND "pulmonary"[All Fields] AND "disease"[All Fields])) AND ("acute"[All Fields] OR "acutely"[All Fields] OR "acutes"[All Fields]) AND ("exacerbate"[All Fields] OR "exacerbated"[All Fields] OR "exacerbates"[All Fields] OR "exacerbating"[All Fields] OR "exacerbation"[All Fields] OR "exacerbations"[All Fields] OR "exacerbator"[All Fields] OR "exacerbators"[All Fields])) OR ("asthma"[MeSH Terms] OR "asthma"[All Fields] OR "asthmas"[All Fields] OR "asthma s"[All Fields]) OR (("asthma"[MeSH Terms] OR "asthma"[All Fields] OR ("bronchial"[All Fields] AND "asthma"[All Fields]) OR "bronchial asthma"[All Fields]) AND ("acute"[All Fields] OR "acutely"[All Fields] OR "acutes"[All Fields]) AND ("exacerbate"[All Fields] OR "exacerbated"[All Fields] OR "exacerbates"[All Fields] OR "exacerbating"[All Fields] OR "exacerbation"[All Fields] OR "exacerbations"[All Fields] OR "exacerbator"[All Fields] OR "exacerbators"[All Fields])) OR ("cough"[MeSH Terms] OR "cough"[All Fields] OR "coughing"[All Fields] OR "coughs"[All Fields] OR "coughed"[All Fields]) OR (("cough"[MeSH Terms] OR "cough"[All Fields] OR "coughing"[All Fields] OR "coughs"[All Fields] OR "coughed"[All Fields]) AND ("infectious"[All Fields] OR "infectiousness"[All Fields]) AND ("acute"[All Fields] OR "acutely"[All Fields] OR "acutes"[All Fields])) OR "pulmonary disease, chronic obstructive"[MeSH Terms] OR ((("chronic"[All Fields] OR "chronical"[All Fields] OR "chronically"[All Fields] OR "chronicities"[All Fields] OR "chronicity"[All Fields] OR "chronicization"[All Fields] OR "chronics"[All Fields]) AND ("obstruct"[All Fields] OR "obstructed"[All Fields] OR "obstructing"[All Fields] OR "obstruction"[All Fields] OR

"obstructions"[All Fields] OR "obstructive"[All Fields] OR "obstructs"[All Fields]) AND ("lung"[MeSH Terms] OR "lung"[All Fields] OR "pulmonary"[All Fields])) AND "acute disease"[MeSH Terms]) OR "asthma chronic obstructive pulmonary disease overlap syndrome"[MeSH Terms] OR "asthma"[MeSH Terms] OR "cough"[MeSH Terms] OR "chronic cough"[MeSH Terms])

Web of scienc
(AECOPD) or (COPD) or (Asthma) or (Bronchial Asthma, Acute Exacerbation) or (Cough, Infectious, Acute) or (cough) (All Fields) and ((Qingke Pingchuan) or (Qingke Pingchuan Granules)) (All Fields)

Embase
((acute exacerbation of chronic obstructive pulmonary disease OR aecopd OR copd) OR (acute exacerbation of bronchial asthma OR bronchial asthma, acute exacerbation OR (bronchial AND asthma AND acute AND exacerbation)) OR (acute bronchitis OR (acute AND bronchitis)) OR (acute infectious cough OR (cough AND infectious AND acute)))AND("qingke pingchuan granules" OR "qingke pingchuan")

Cochrane Library
Qingke Pingchuan or Qingke Pingchuan Granule in Title Abstract Keyword AND (AECOPD) or (COPD) or (Asthma) or (Bronchial Asthma, Acute Exacerbation) or (Cough, Infectious, Acute) or (cough) in Title Abstract Keyword - (Word variations have been searched)

CNKI
(主题：慢性阻塞性肺疾病急性加重+AECOPD+慢阻肺+COPD+支气管哮喘急性加+哮喘+急性咳嗽+咳嗽+支气管炎(精确))AND(主题：清咳平喘颗粒(精确))

VIP
题名或关键词:(慢性阻塞性肺疾病急性加重 or AECOPD or 慢阻肺 or COPD or 支气管哮喘急性加重 or 哮喘 or 急性咳嗽 or 咳嗽 or 支气管炎) and 题名或关键词:("清咳平喘颗粒")

WanFang
主题:(慢性阻塞性肺疾病急性加重 or AECOPD or 慢阻肺 or COPD or 支气管哮喘急性加重 or 哮喘 or 急性咳嗽 or 咳嗽 or 支气管炎) and 主题:("清咳平喘颗粒")

Supplementary table2. Formulas for Estimating Standard Deviation (SD) in Studies with Different Data Reporting Formats

For studies reporting median and interquartile range	SD = IQR / 1.35
For studies reporting only pre-treatment and post-treatment values	SD = $\sqrt{(SD1^2 + SD2^2 - 2 \times r \times SD1 \times SD2)}$, r defaults to 0.5

supplementary Figure：

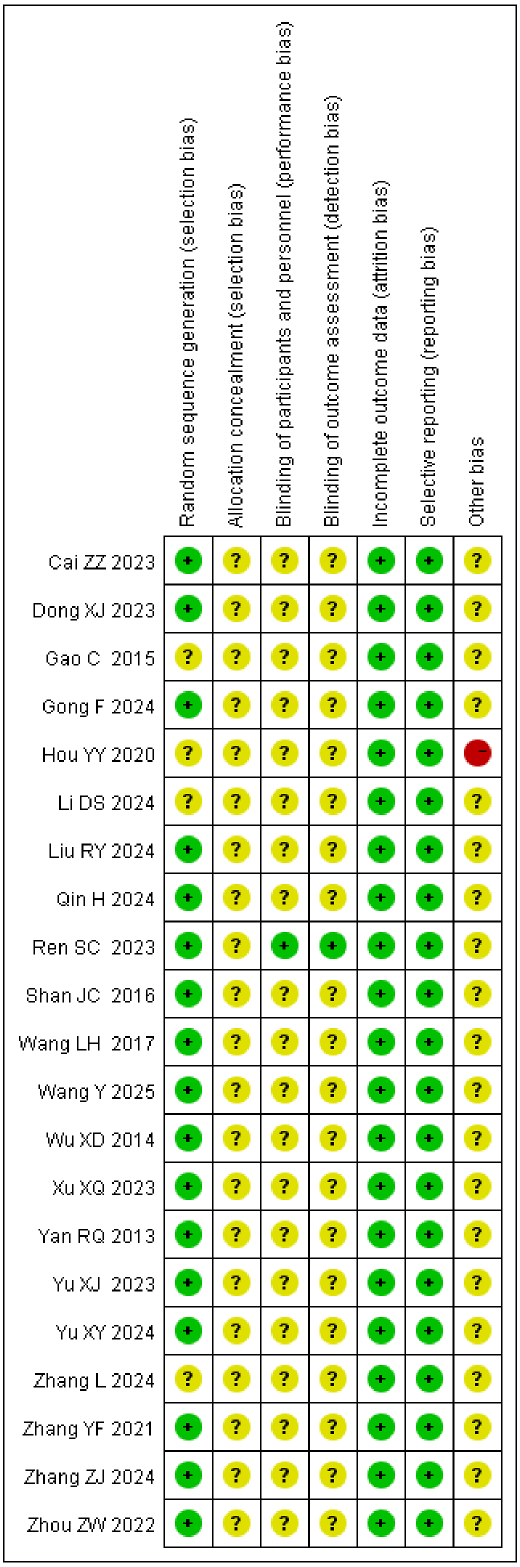

supplementary Figure 1. Risk of bias summary

Study or Subgroup	Experimental Events	Experimental Total	Control Events	Control Total	Weight	Risk Ratio M-H, Fixed, 95% CI
**1.2.1 1**						
Cai ZZ 2023	37	40	32	40	4.0%	1.16 [0.97, 1.38]
Dong XJ 2023	30	30	26	30	3.3%	1.15 [0.99, 1.34]
Gao C 2015	30	30	28	30	3.5%	1.07 [0.96, 1.20]
Li DS 2024	37	40	30	40	3.7%	1.23 [1.01, 1.51]
Liu RY 2024	49	50	43	50	5.3%	1.14 [1.01, 1.28]
Qin H 2024	88	90	79	88	9.9%	1.09 [1.01, 1.18]
Wang LH 2017	30	32	24	32	3.0%	1.25 [1.00, 1.56]
Xu XQ 2023	48	50	45	50	5.6%	1.07 [0.96, 1.19]
Yu XJ 2023	70	80	64	80	7.9%	1.09 [0.95, 1.25]
Zhang ZJ 2024	47	50	42	50	5.2%	1.12 [0.97, 1.29]
Zhou ZW 2022	45	50	40	50	5.0%	1.13 [0.95, 1.33]
**Subtotal (95% CI)**		**542**		**540**	**56.5%**	**1.12 [1.08, 1.17]**
Total events	511		453			
Heterogeneity: Chi² = 4.35, df = 10 (P = 0.93); I² = 0%						
Test for overall effect: Z = 5.39 (P < 0.00001)						
**1.2.2 2**						
Gong F 2024	64	68	53	68	6.6%	1.21 [1.05, 1.39]
Hou YY 2020	29	30	22	30	2.7%	1.32 [1.05, 1.65]
Shan JC 2016	53	60	42	60	5.2%	1.26 [1.04, 1.53]
Wu XD 2014	77	80	69	80	8.6%	1.12 [1.01, 1.23]
Yan RQ 2013	46	50	41	50	5.1%	1.12 [0.96, 1.31]
Yu XY 2024	47	48	44	47	5.5%	1.05 [0.96, 1.14]
Zhang L 2024	47	48	39	45	5.0%	1.13 [1.00, 1.28]
Zhang YF 2021	48	50	39	50	4.8%	1.23 [1.05, 1.44]
**Subtotal (95% CI)**		**434**		**430**	**43.5%**	**1.17 [1.11, 1.23]**
Total events	411		349			
Heterogeneity: Chi² = 10.02, df = 7 (P = 0.19); I² = 30%						
Test for overall effect: Z = 5.99 (P < 0.00001)						
**Total (95% CI)**		**976**		**970**	**100.0%**	**1.14 [1.11, 1.18]**
Total events	922		802			
Heterogeneity: Chi² = 14.45, df = 18 (P = 0.70); I² = 0%						
Test for overall effect: Z = 8.03 (P < 0.00001)						
Test for subgroup differences: Chi² = 1.24, df = 1 (P = 0.26), I² = 19.5%						

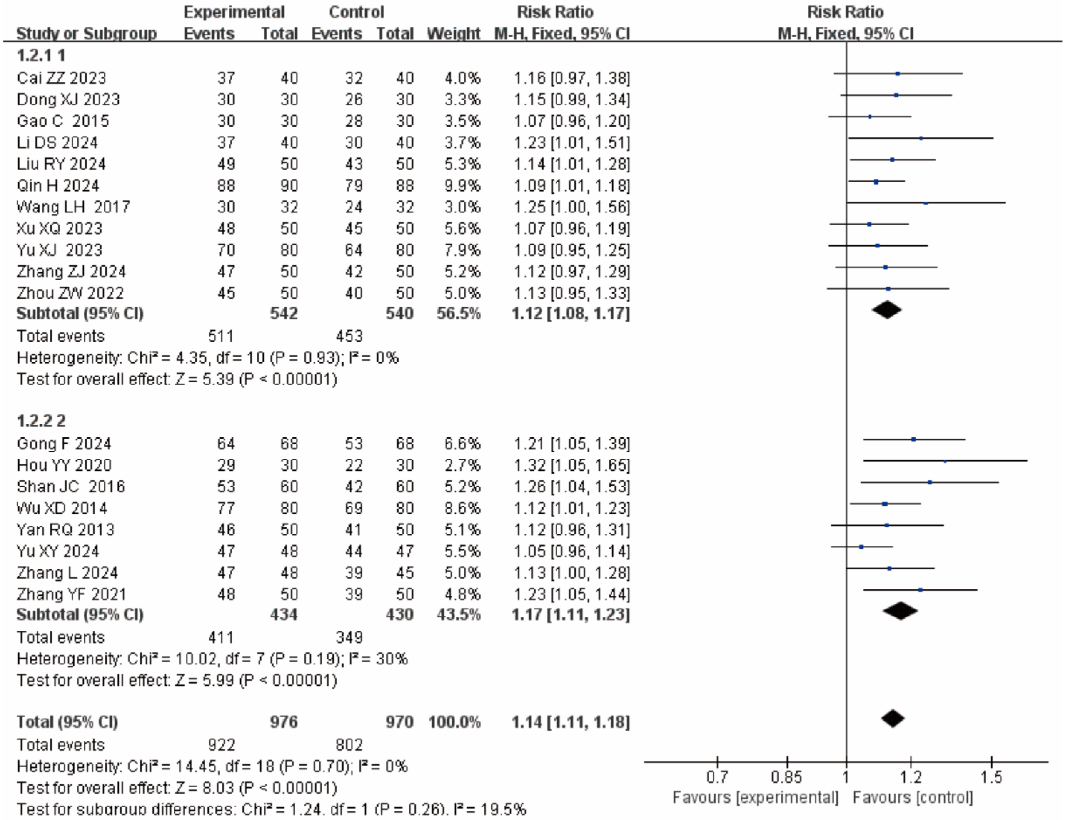

Effective rate

Study or Subgroup	Experimental Mean	Experimental SD	Experimental Total	Control Mean	Control SD	Control Total	Weight	Mean Difference IV, Random, 95% CI
**9.3.1 1**								
Dong XJ 2023	13.35	6.96	30	10.23	6.91	30	14.2%	3.12 [-0.39, 6.63]
Gao C 2015	4.06	7.85	30	3.78	6.72	40	14.3%	0.28 [-3.22, 3.78]
Li DS 2024	11.68	10.04	40	4.87	9.85	30	11.6%	6.81 [2.11, 11.51]
Liu RY 2024	14.38	11.25	50	8.3	10.05	50	12.7%	6.08 [1.90, 10.26]
**Subtotal (95% CI)**			**150**			**150**	**52.8%**	**3.81 [0.88, 6.74]**
Heterogeneity: Tau² = 4.89; Chi² = 6.67, df = 3 (P = 0.08); I² = 55%								
Test for overall effect: Z = 2.55 (P = 0.01)								
**9.3.2 2**								
Wang Y 2025	16.19	5.99	41	7.22	5.97	41	16.3%	8.97 [6.38, 11.56]
Wu XD 2014	7.06	10.46	80	2.79	12.18	80	14.2%	4.27 [0.75, 7.79]
Zhang YF 2021	13	7.08	50	11	5.37	50	16.6%	2.00 [-0.46, 4.46]
**Subtotal (95% CI)**			**171**			**171**	**47.2%**	**5.09 [0.65, 9.53]**
Heterogeneity: Tau² = 13.25; Chi² = 14.88, df = 2 (P = 0.0006); I² = 87%								
Test for overall effect: Z = 2.25 (P = 0.02)								
**Total (95% CI)**			**321**			**321**	**100.0%**	**4.45 [1.96, 6.94]**
Heterogeneity: Tau² = 8.13; Chi² = 23.09, df = 6 (P = 0.0008); I² = 74%								
Test for overall effect: Z = 3.51 (P = 0.0005)								
Test for subgroup differences: Chi² = 0.22, df = 1 (P = 0.64), I² = 0%								

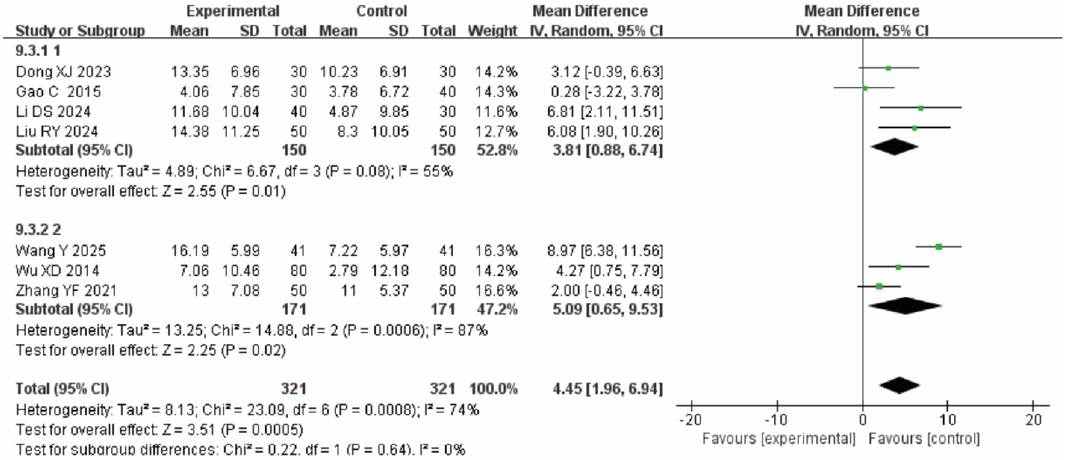

FEV1%

supplementary Figure 2. Subgroup Analysis by TCM Syndrome Types

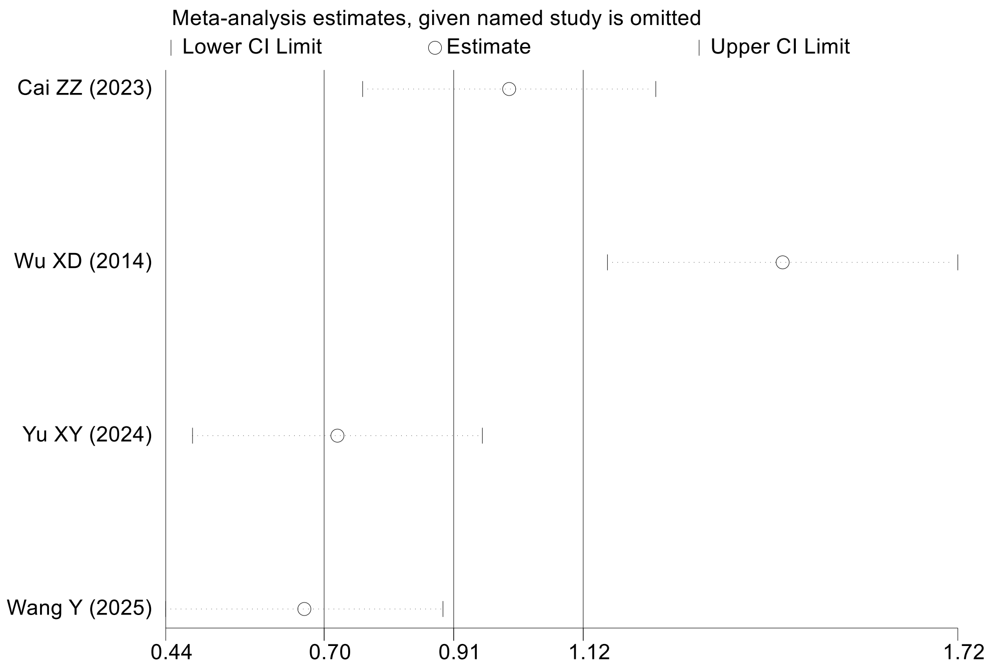

supplementary Figure 3. sensitivity analysis of PaO_2.

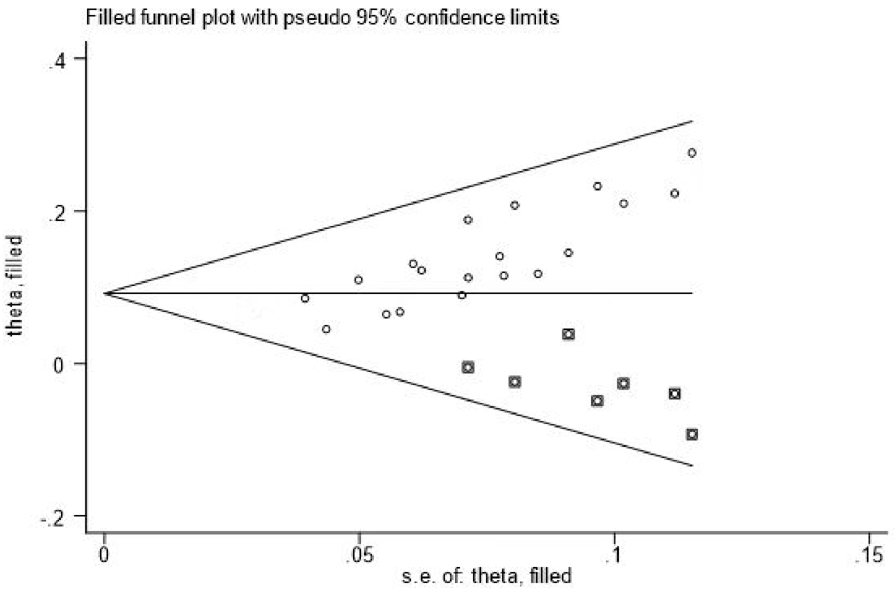

supplementary Figure 4. Trim-and-fill analysis for Effeacy rate and Time to cough resolution.

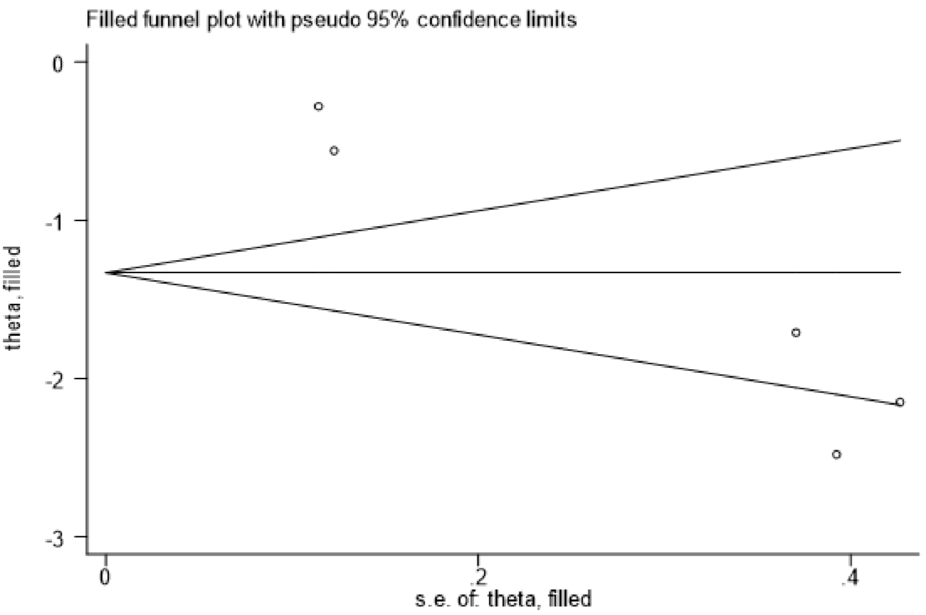

supplementary Figure 5. Trim-and-fill analysis for Time to cough resolution.

Supplementary table3. Summary of Conventional Interventions in Included Studies

Study	Conventional Intervention	Study	Conventional Intervention
Cai ZZ 2023	Anti-infection, anti-inflammation, relieving spasm and asthma, relieving cough, expectorant therapy, and oxygen therapy	Zhou ZW 2022	Symptomatic treatment and Salmeterol Xinafoate and Fluticasone Propionate Dry Powder Inhalation
Li DS 2024	Oxygen inhalation, anti-infection, anti-inflammation, relieving spasm and asthma, relieving cough and eliminating phlegm	Dong XJ 2023	Symptomatic treatment, Budesonide Suspension for Inhalation, Terbutaline Sulfate Nebulizing Solution
Liu RY 2024	Oxygen inhalation, relieving cough and asthma, expectorant therapy, and anti-infection treatment	Gong F 2024	Symptomatic treatment and Fluticasone Propionate Inhalation Aerosol
Ren SC 2023	Oxygen therapy, relieving cough and eliminating phlegm, relieving spasm and asthma, and antibiotic therapy	Hou YY 2020	Symptomatic treatment, Levofloxacin Hydrochloride Tablets, Ambroxol Hydrochloride Tablets, Aminophylline Tablets, and cupping therapy
Wu XD 2014	Smoking cessation, Cephalosporins, Ambroxol Hydrochloride, Aminophylline; oxygen inhalation; correcting electrolyte disorders and acid-base imbalance	Shan JC 2016	Symptomatic treatment, antipyretic therapy, and Cefixime Granules
Yu XY 2024	Oxygen inhalation, relieving cough and eliminating phlegm, relieving	Yan RQ 2013	Symptomatic treatment and Ambroxol Hydrochloride Oral Solution

	asthma, and anti-infection		
Zhang ZJ 2024	Oxygen inhalation, Long-acting Anticholinergics (LAMAs), Long-acting β2 Agonists (LABAs), Inhaled Corticosteroids (ICS); antibiotics; mechanical ventilation	Zhang L 2024	Symptomatic treatment, oxygen inhalation, antipyretic therapy, and Amoxicillin Capsules
Wang Y 2025	Oxygen inhalation, expectorant and anti-asthmatic therapy, antibiotics, nebulization with Ipratropium Bromide Solution and Terbutaline Sulfate Nebulizing Solution	Yu XJ 2023	Symptomatic treatment, Cefaclor for Suspension, and Ambroxol Hydrochloride Oral Solution
Wang LH 2017	Antibiotics for infection prevention, relieving spasm, expectorant therapy, oxygen therapy,	Qin H 2024	Symptomatic treatment and Compound Methoxyphenamine Capsules
Gao C 2015	Routine supportive care, Terbutaline Sulfate Nebulizing Solution, and Budesonide Suspension for Inhalation	Xu XQ 2023	Supportive therapy, antihistamines, inhaled or oral corticosteroids, and Leukotriene Receptor Antagonists (LTRAs)
Zhang YF 2021	Oxygen inhalation, Cefotiam, Budesonide, and Terbutaline nebulization		
